# Integrative In Silico Multi-Omics Profiling of circRNA-Mediated ceRNA Networks Reveals Prognostic Biomarkers and Repurposed Therapeutic Candidates in Gastric Cancer

**DOI:** 10.3390/ijms27052171

**Published:** 2026-02-25

**Authors:** Melike Ebrar Bakirci, Busra Aydin

**Affiliations:** 1 Department of Biotechnology, Institute of Postgraduate Education, Konya Food and Agriculture University, Konya 42090, Turkey; 233012010001@ogr.gidatarim.edu.tr; 2 Department of Bioengineering, Faculty of Engineering and Architecture, Konya Food and Agriculture University, Konya 42090, Turkey; 3 Department of Molecular Biology and Genetics, Faculty of Agriculture and Natural Sciences, Konya Food and Agriculture University, Konya 42090, Turkey

**Keywords:** transcriptomics, gastric cancer, circRNA, miRNA, ceRNA network, molecular docking, drug repositioning, biomarkers

## Abstract

Gastric cancer (GC), also known as stomach adenocarcinoma (STAD), remains a highly lethal malignancy due to late diagnosis, limited therapeutic efficacy, and frequent metastasis. Although extensive molecular profiling has been performed, post-transcriptional regulatory mechanisms underlying GC progression are still incompletely characterized. In this study, we applied an integrative multi-omics framework to elucidate the regulatory roles and clinical relevance of circular RNAs (circRNAs) in GC. Transcriptomic data of mRNAs, microRNAs, and circRNAs from eight independent GEO datasets were jointly analyzed, resulting in the identification of 249 differentially expressed genes (DEGs), 8 differentially expressed microRNAs (DEmiRNAs), and 4 differentially expressed circRNAs (DEcircRNAs). These molecules were integrated into a competing endogenous RNA (ceRNA) network, enabling systems-level characterization of GC-associated regulatory interactions. Network topology and survival analyses prioritized 13 hub molecules, including *IGF2BP3*, *COL4A1*, *MMP14*, and *TGM2*, which showed both central network positions and significant associations with patient survival. To explore therapeutic implications, transcriptomics-guided drug repositioning combined with molecular docking analysis identified five candidate compounds—celastrol, fedratinib, pevonedistat, tozasertib, and withaferin A—predicted to target key network hubs. Overall, this in silico study provides a ceRNA-centered regulatory framework for GC and prioritizes biologically informed biomarkers and repositioned drug candidates with potential applicability across other malignancies to converge precision oncology.

## 1. Introduction

Gastric cancer (GC), also referred to as stomach cancer or stomach adenocarcinoma (STAD), remains one of the leading causes of cancer-related mortality worldwide despite declining incidence in some regions due to improved hygiene, *Helicobacter pylori* eradication, and dietary changes [1]. Globally, GC is the fifth most common malignancy and the fourth leading cause of cancer-related deaths, accounting for over 770,000 deaths annually [2]. In 2022, more than 1.1 million new cases of GC were diagnosed [3]. The most common histological subtype of GC is gastric adenocarcinoma, particularly of the intestinal and diffuse types, which together represent over 90% of all cases [4]. Early diagnosis is challenging due to nonspecific symptoms, and many cases are diagnosed at advanced or metastatic stages, contributing to the disease’s poor prognosis [5].

Histopathologically, gastric adenocarcinomas are classified into several subtypes according to the World Health Organization (WHO), including tubular, papillary, mucinous, poorly cohesive (including signet-ring cell carcinoma), and mixed adenocarcinomas [6]. Molecular heterogeneity and treatment resistance also complicate management and reduce survival rates. Despite the availability of chemotherapy, targeted agents, and immunotherapies, five-year survival remains below 40%, especially in advanced-stage disease.

In recent years, non-coding RNAs (ncRNAs)—particularly circular RNAs (circRNAs)—have emerged as critical post-transcriptional regulators in cancer biology. circRNAs are characterized by a covalently closed-loop structure, lacking both 5′ caps and 3′ poly(A) tails, which renders them highly stable compared to linear RNAs [7,8]. This stability allows circRNAs to act as microRNA (miRNA) sponges, effectively sequestering miRNAs and preventing them from binding to their mRNA targets, thereby modulating gene expression. For instance, circNRIP1 has been reported to promote GC progression by targeting miR-149-5p/AKT1 signaling, while circRNA_100269 acts as a tumor suppressor through its interaction with miR-630 [9]. These interactions contribute to the establishment of a competing endogenous RNA (ceRNA) network, wherein circRNAs, miRNAs, and mRNAsform intricate regulatory loops that drive tumorigenesis and metastasis [10].

Although significant efforts have been made to elucidate the molecular mechanisms underlying GC and various targeted therapies and small molecules have been proposed, effective clinical translation remains limited. There is a critical need for omics-based approaches that can uncover reliable biomarkers and therapeutic targets. While some advancements have been achieved through genomic and transcriptomic profiling [11,12,13], there is still a notable gap in personalized treatment strategies and clinical validation.

In this in silico study, we performed an integrative transcriptomic analysis of GC by comparing microarray datasets from GC patients and healthy controls to identify differentially expressed circRNAs (DEcircRNAs), miRNAs (DEmiRNAs), and mRNAs (DEGs). Subsequently, we constructed a comprehensive ceRNA regulatory network (DEcircRNA–DEmiRNA–DEG) specific to GC and identified novel hub gene–drug interactions. Potential repositioned drugs were prioritized, and molecular docking analysis was used to validate their binding capacity in silico. This investigation aims to provide novel insights into the ceRNA-mediated gene regulatory landscape in GC and offer promising biomarkers and therapeutic candidates for future clinical translation.

## 2. Results

### 2.1. Identification and Functional Characterization of DEGs, DEmiRNAs, and DEcircRNAs in Disease-Associated Transcriptomic Profiles

**Figure 1:**
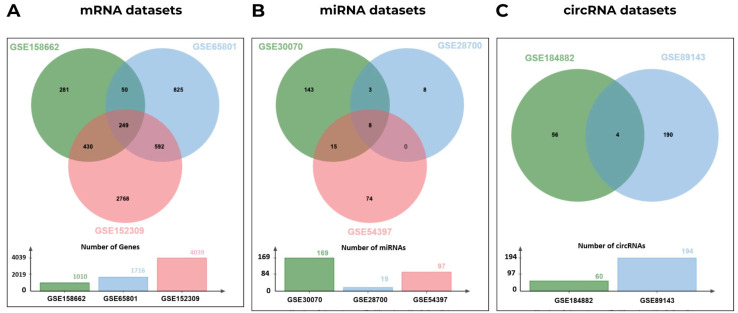
Venn diagrams illustrating the overlap among datasets: (**A**) mRNA datasets, (**B**) miRNA datasets, and (**C**) circRNA datasets.

A total of 249 differentially expressed genes (DEGs) were identified through integrative analysis of three transcriptomic datasets (GSE158662, GSE65801, and GSE152309). Differential expression analysis of microRNA profiles (GSE30070, GSE28700, and GSE54397) revealed 8 significantly altered microRNAs (DEmiRNAs), while 4 differentially expressed circular RNAs (DEcircRNAs) were obtained from two circRNA expression datasets (GSE184882 and GSE89143) under the predefined threshold conditions (Figure 1). Although multiple independent datasets were used rather than a single matched cohort, standardized normalization procedures, intersection-based DEG selection, and multi-level validation strategies were employed to minimize inter-dataset heterogeneity and enhance the robustness and generalizability of the findings.

**Figure 2:**
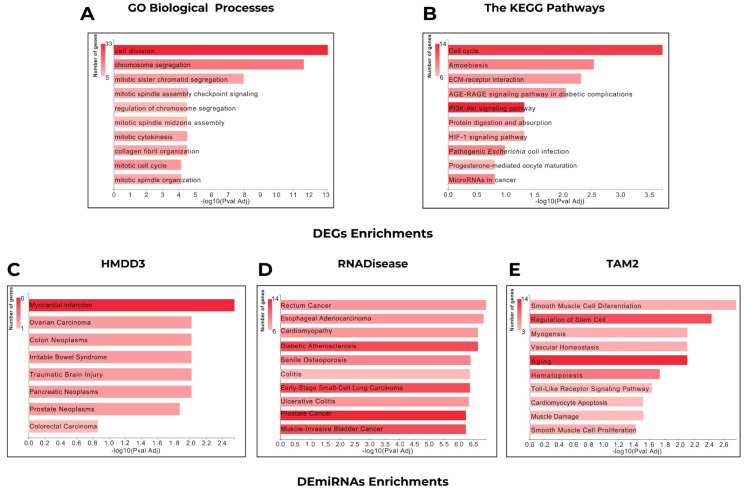
Functional enrichment analysis of overlapping (**A**,**B**) DEGs and (**C**–**E**) DEmiRNAs.

Gene Ontology (GO) enrichment analysis revealed significant functional associations primarily related to cell cycle regulation, mitotic processes, and extracellular matrix organization (Figure 2A). Pathway enrichment analysis of the DEGs revealed significant involvement in key oncogenic and cellular regulatory pathways. These included the cell cycle, *PI3K-Akt* signaling pathway, microRNAs in cancer, pathogenic *Escherichia coli* infection, amoebiasis, and ECM-receptor interaction pathways (Figure 2B). These results suggest a multifaceted molecular involvement in both infectious and proliferative disease processes. These findings are indicative of perturbations in the cytoskeletal and extracellular matrix (ECM) dynamics, with potential implications in tissue remodeling and tumor progression.

Further analysis of DEmiRNAs using the HMDD v3.0 database revealed functional associations with myocardial interactions, colon neoplasms, and pancreatic neoplasms, emphasizing the pathological relevance of the altered microRNA profiles in various cancer types (Figure 2C). RNADisease analysis further supported the implication of these miRNAs in head and neck cancer, pancreatic neoplasm, colitis, rectum cancer, esophageal adenocarcinoma, endometriosis, and senile osteoporosis (Figure 2D).

These associations underline a broad spectrum of disease relevance, spanning from inflammatory to degenerative and malignant conditions. TAM2-based functional annotation of the identified DEmiRNAs provided further insights, indicating significant enrichment in pathways related to aging, regulation of stem cell functions, hematopoiesis, tumor suppressor microRNAs, and lipid metabolism (Figure 2E). These results collectively suggest that the miRNA dysregulation may contribute to both age-related degenerative processes and cancer-associated molecular mechanisms.

### 2.2. Visualization of ceRNA Network

**Figure 3:**
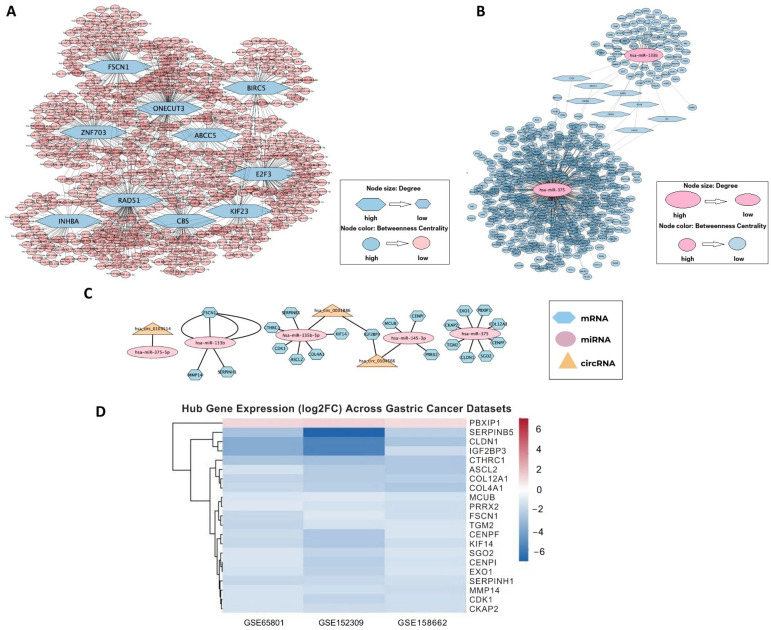
Integrated regulatory networks illustrating (**A**) DEG–miRNA interactions, (**B**) DEmiRNA–mRNA regulatory relationships, and (**C**) the comprehensive DEG–DEmiRNA–DEcircRNA interaction network (ceRNA network). The node size represents degree centrality, and color intensity represents betweenness centrality of the network elements. (**D**) Heatmap illustrating log2 Fold Change expression levels of hub genes across three independent gastric cancer transcriptomics datasets (GSE65801, GSE152309, and GSE158662). Rows represent hub genes, and columns correspond to individual datasets. Color intensity reflects relative expression changes between gastric cancer and normal samples, with blue indicating downregulation and red indicating upregulation.

To investigate the post-transcriptional regulatory landscape and the ceRNA crosstalk in the studied context, a series of interaction networks was constructed based on integrative expression analyses of 249 DEGs, 8 DEmiRNAs, and 4 DEcircRNAs.The DEGs-miRNA interaction network was established to illustrate the interactions between DEGs and their validated miRNA regulators using experimentally confirmed data from the miRTarBase database. (Figure 3A). This DEG–miRNA interaction network comprised 761 nodes and 970 edges, reflecting a complex transcriptional landscape. Topological analysis based on degree and betweenness centrality identified several hub molecules with potential central regulatory roles, including *FSCN1*, *ZNF703*, *ONECUT3*, *ABCC5*, *BIRC5*, *E2F3*, *KIF23*, *CBS*, *RAD51*, *and INHBA*. These hub molecules may play pivotal roles in coordinating gene expression programs associated with the underlying pathophysiological mechanisms (Table 1).

The DemiRNAs-target gene interaction network was derived by mapping DEmiRNAs to their putative target genes, thereby capturing the direct regulatory influence of miRNAs on gene expression (Figure 3B). This miRNA–mRNA network revealed 555 nodes and 761 edges. Among the DEmiRNAs, hsa-miR-375 and hsa-miR-133b were observed to regulate a considerable number of downstream DEGs, indicating their central roles in transcriptomic modulation. These miRNAs potentially serve as master regulators in the disease-relevant miRNA–mRNA interaction network.

To further refine the ceRNA landscape and delineate post-transcriptional regulatory axes involving circRNAs, a core ceRNA interaction network (DEGs–DEmiRNAs–DEcircRNAs) was constructed by integrating hub components from the previous DEG–miRNA and miRNA–mRNA networks with DEcircRNAs (Figure 3C). The resulting ceRNA core network consisted of 29 nodes and 25 edges, representing a focused subnetwork that highlights circRNA–miRNA–mRNA regulatory relationships.

This network incorporated several miRNAs frequently implicated in cancer-related regulatory processes, including *hsa-miR-375*, *hsa-miR-133b*, *hsa-miR-133b-3p*, *hsa-miR-375-5p*, *hsa-miR-145*, and *hsa-miR-145-3p*, suggesting their recurrent involvement in ceRNA-mediated regulation within the constructed network. In addition, three circRNAs—*hsa_circ_0001886*, *hsa_circ_0104566*, and *hsa_circ_0103504*—were identified as upstream components participating in miRNA-associated regulatory interactions.

Specifically, IGF2BP3 was linked to two circRNAs through shared miRNAs, forming circRNA–miRNA–IGF2BP3 regulatory axes consistent with the ceRNA hypothesis. These interactions suggest potential competitive endogenous RNA mechanisms whereby circRNAs may influence IGF2BP3 expression by competing for common miRNA binding sites. Within the ceRNA network, the association between circRNAs and IGF2BP3 reflects miRNA-mediated regulatory relationships rather than direct physical interactions.

Heatmap visualization demonstrated consistent downregulation of the majority of hub genes across all three independent mRNA datasets, supporting the robustness of the identified transcriptional alterations. PBXIP1 was the only gene showing consistent upregulation across cohorts.

Collectively, the integration of these regulatory layers provides a structured ceRNA framework that illustrates complex RNA–RNA interaction patterns and identifies candidate regulatory relationships for further functional investigation.

### 2.3. Drug Repositioning Analysis Based on Hub Gene Signatures

To identify candidate therapeutic compounds capable of reversing disease-specific gene expression patterns, drug repositioning analysis was performed using the L1000CDS^2^ platform based on the fold-change profiles of the 21 hub molecules. Compounds were ranked according to the “1—cosine” similarity score, which quantifies the inverse correlation between the disease-associated transcriptional signature and the drug-induced gene expression profile, with higher scores indicating a stronger reversal potential.

The initial screening yielded 50 small-molecule compounds predicted to counteract the disease-related expression pattern. Following further prioritization based on mechanism of action, FDA approval status, clinical indications, and documented antineoplastic relevance, 12 compounds were shortlisted for detailed evaluation. Among these, five compounds—Withaferin A, Tozasertib, Pevonedistat, Fedratinib, and Celastrol—exhibited consistently higher L1000CDS2 scores, indicating a stronger and more reproducible reverse transcriptional effect.

Of these prioritized compounds, Pevonedistat, Tozasertib, Withaferin A, and Celastrol are currently under clinical or preclinical investigation, while Fedratinib is an FDA-approved drug for other clinical indications. The L1000CDS^2^ score values, together with pharmacological profiles and regulatory status of the selected compounds, are summarized in Table 2, providing quantitative support for their potential antineoplastic relevance and therapeutic repositioning.

### 2.4. Prognostic Value Assessment of Hub Molecules

**Figure 4:**
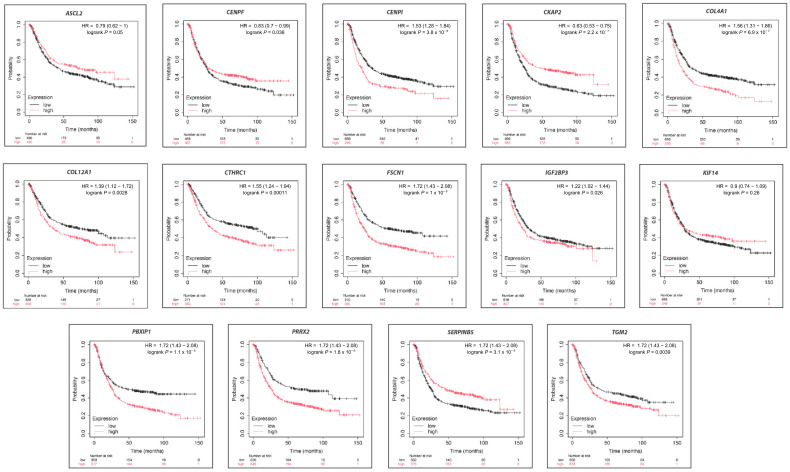
Survival analysis highlighting the prognostic relevance of hub molecules, including statistical significance, was determined by a log-rank *p*-value < 0.05 regarding overall survival.

To evaluate the prognostic relevance of the identified hub molecules, Kaplan–Meier (KM) survival analysis was conducted using the KM-Plotter tool, which integrates high-throughput gene expression data and survival outcomes from multiple publicly available datasets, including Gene Expression Omnibus (GEO), European Genome-Phenome Archive (*EGA*), and The Cancer Genome Atlas (TCGA). Patients were stratified into high- and low-expression groups based on expression quantiles, and overall survival differences were assessed using log-rank tests (Figure 4). Among the analyzed genes, 13 hub molecules showed a statistically significant association with patient survival, including *IGF2BP3*, *COL4A1*, *KIF14*, *MCUB*, *CENPI*, *PRRX2*, *CLDN1*, *MMP14*, *CKAP2*, *FSCN1*, *SERPINH1*, *SGO2*, and *TGM2.* Notably, these hub molecules exhibited heterogeneous prognostic patterns. Elevated expression levels of *COL4A1*, *CENPI*, *COL12A1*, *TGM2*, *PRRX2*, *PBXIP1*, *IGF2BP3*, *FSCN1,* and *CTHRC1* were associated with poorer overall survival, as reflected by hazard ratios greater than 1.

In contrast, higher expression of *SERPINB5*, *CKAP2*, *ASCL2*, *KIF14*, and *CENPF* was associated with more favorable survival outcomes, suggesting context-dependent or protective roles for these genes in gastric cancer. Importantly, these prognostic trends were not always concordant with the differential expression patterns observed in the original cDNA microarray datasets, indicating that the impact of hub molecule expression on patient survival may depend on biological context rather than expression level alone.

### 2.5. Molecular Docking Analysis with Candidate-Repositioned Drugs

**Figure 5:**
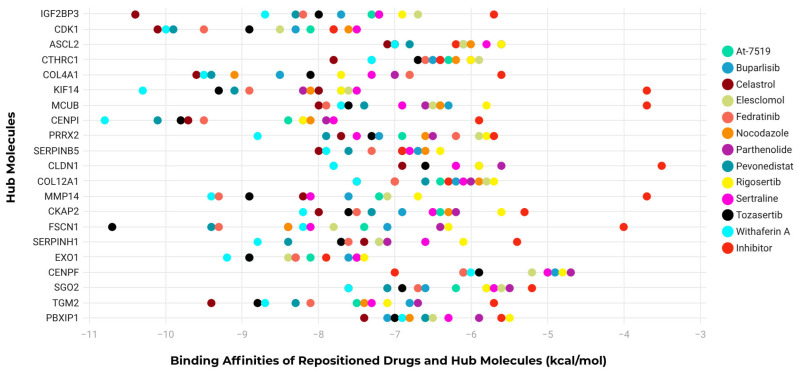
Molecular docking analysis depicting the binding affinities of ligands, including hub inhibitors and repositioned drug candidates. Inhibitors of each ligand were used as positive controls to compare the effectiveness of the binding. Distinct colors indicate individual compounds.

Molecular docking analysis was performed to evaluate the interactions between the protein products encoded by the 21 core genes and 12 repurposed drugs, along with one inhibitor (Figure 5). Docking-derived binding energies were calculated for each protein–compound pair and converted into ratio scores to enable comparative assessment across the full set of target proteins. Based on this analysis, Withaferin A, Celastrol, Fedratinib, Pevonedistat, and Tozasertib consistently exhibited favorable docking scores across multiple targets, indicating stable in silico interactions.

In parallel, a subset of protein products showed stronger, more consistent docking scores with the selected compounds and were therefore defined as druggable targets in the docking-based analysis. These targets included *IGF2BP3*, *COL4A1*, *KIF14*, *MCUB*, *CENPI*, *PRRX2*, *CLDN1*, *MMP14*, *CKAP2*, *FSCN1*, *SERPINH1*, *SGO2*, and *TGM2.* The utilized tool, CB-Dock, performs blind docking and identifies optimal ligand-binding cavities without restricting the search to known catalytic sites. The predicted interactions were interpreted as exploratory and hypothesis-generating. Binding to non-catalytic cavities may reflect potential allosteric modulation rather than direct enzymatic inhibition, and therefore, functional effects cannot be inferred solely from docking results. Overall, the docking results highlight five compounds with reproducible interaction profiles across multiple protein targets characterized by comparatively higher docking affinities within this structure-based framework.

### 2.6. ROC Curve Analysis Revealed Predictive Power of Hub Molecules

Receiver operating characteristic (ROC) curve analysis was performed using the ROC Plotter platform (https://rocplot.com/, access date: 24 September 2025) to assess the predictive value of selected hub molecules in the context of drug response in gastric cancer. Unlike conventional diagnostic ROC analyses, this approach was specifically applied to evaluate whether the expression levels of candidate genes could discriminate between responder and non-responder gastric cancer cell line samples following treatment with the selected repositioned drugs.

The ROC Plotter platform integrates gene expression profiles derived from drug-treated cancer cell lines and enables stratification of samples based on therapeutic response. Accordingly, the area under the curve (AUC) was used as a quantitative measure of the ability of individual hub molecules to predict treatment-associated transcriptional differences, where higher AUC values indicate stronger discriminatory performance between response groups. Importantly, this analysis does not directly assess drug efficacy, but rather supports the potential utility of the identified hub molecules as predictive biomarkers associated with drug responsiveness at the transcriptomic level.

**Figure 6:**
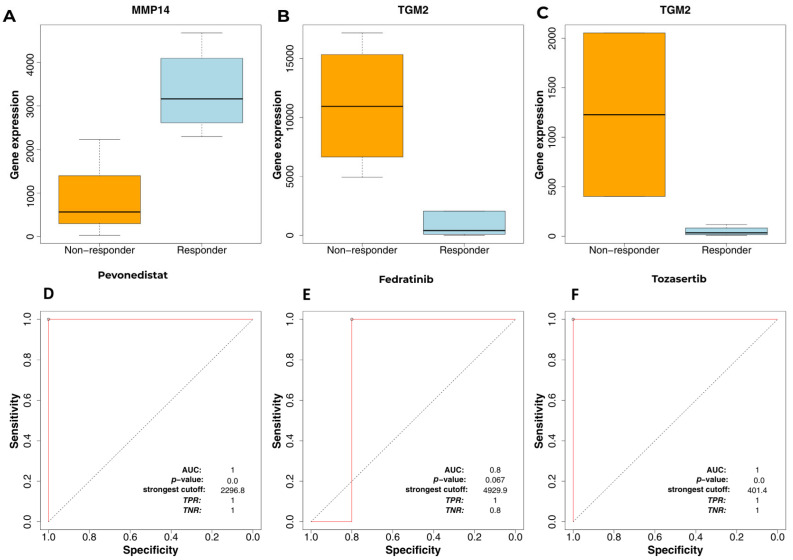
Predictive ROC analysis of hub molecules in drug-treated gastric cancer models. Expression patterns and ROC curve analyses of *MMP14* and *TGM2*, the two hub molecules exhibiting the strongest predictive performance in the drug-response context. (**A**–**C**) Upper panels show differential gene expression levels between responder and non-responder gastric cancer cell line samples following treatment with pevonedistat (**A**), fedratinib (**B**), and tozasertib (**C**), respectively. The solid lines inside the bars represented the median values. (**D**–**F**) Lower panels display the corresponding receiver operating characteristic (ROC) curves evaluating the ability of *MMP14* and *TGM2* expression levels to discriminate between responder and non-responder groups. The area under the curve (AUC) values indicate the predictive strength of each gene in relation to drug response.The dashed diagonal line represents the reference line of no discrimination (AUC = 0.5), indicating random classifier performance in ROC analysis.

The ROC curve analyses demonstrated the discriminatory potential of the identified hub molecules in the context of drug response in gastric cancer (Figure 6A–C). While most hub molecules exhibited measurable predictive performance, *MMP14* and *TGM2* consistently showed the highest AUC values, indicating a stronger ability to distinguish responder from non-responder samples following treatment. These genes therefore emerged as the most robust predictive biomarkers among the candidates analyzed, with AUC values approaching 1.0 (Figure 6D–F).

Among the five repositioned drug candidates evaluated, only fedratinib, tozasertib, and pevonedistat yielded interpretable and statistically meaningful ROC outputs within the ROC Plotter framework. Accordingly, subsequent analyses focused on these three compounds. The ROC results for these drugs were concordant with the predictive capacity of the 13 hub molecules, and detailed AUC values are provided in Table 3.

Additionally, the ROC curves of *MMP14* and *TGM2*, which exhibited the strongest predictive performance among the analyzed hub molecules, are presented separately in Figure 6.

Collectively, these results support the potential utility of *MMP14* and *TGM2* as predictive biomarkers associated with drug response at the transcriptomic level, further reinforcing the biological relevance of the selected drug candidates within the proposed repurposing framework.

### 2.7. Expression of Key Hub Molecules and Proteins in Gastric Cancer and Normal Tissues

**Figure 7:**
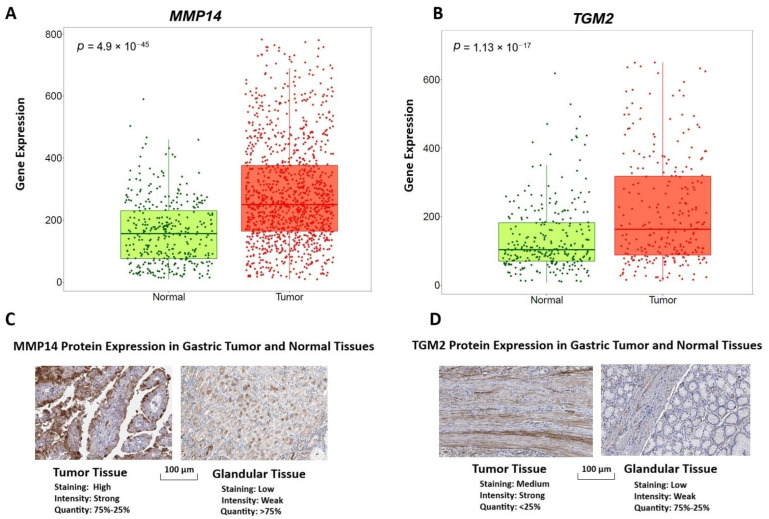
Differential gene and protein expression of MMP14 and TGM2 in gastric cancer and normal tissues. (**A**,**B**) Box plots showing mRNA expression levels of *MMP14* (**A**) and *TGM2* (**B**) in normal gastric tissues and gastric tumor samples, obtained from the TNMplot platform, which integrates GEO, TCGA, and GTEx datasets. The statistical significance between groups is indicated by the corresponding *p*-values. (**C**,**D**) Representative immunohistochemical images of *MMP14* (**C**) and *TGM2* (**D**) protein expression in gastric tumor tissues and normal glandular tissues, retrieved from the Human Protein Atlas database. Staining intensity and the proportion of positively stained cells illustrate increased protein expression in tumor tissues compared to normal counterparts. Quantity represents the proportion of tumor cells exhibiting positive staining. Scale bars represent 100 μm.

Transcriptomic analysis using the TNMplot platform demonstrated that both *MMP14* and *TGM2* were significantly upregulated in gastric cancer tissues compared to normal gastric samples (Figure 7A–D). Metastatic samples were not analyzed as a separate subgroup in this section. *MMP14* exhibited a marked increase in expression in tumor tissues, with a highly significant difference between normal and cancer groups (*p* = 4.9 × 10^−45^). Similarly, *TGM2* expression was significantly elevated in gastric tumors relative to normal tissues (*p* = 1.13 × 10^−17^), indicating consistent transcriptional activation of these genes in the malignant context.

Protein-level validation using Human Protein Atlas data further corroborated the transcriptomic findings. Immunohistochemical staining revealed stronger and more extensive *MMP14* protein expression in gastric tumor tissues compared to normal glandular tissues, where staining was generally moderate and more diffusely distributed. Likewise, *TGM2* protein expression was more prominent in tumor samples, characterized by increased staining intensity, whereas normal glandular tissues showed weaker staining patterns. Together, these results demonstrate concordant upregulation of *MMP14* and *TGM2* at both the gene and protein expression levels, supporting their relevance as key molecular drivers associated with gastric cancer pathology. Future studies incorporating metastatic lesions, sex-stratified cohorts, and matched tumor–normal tissue pairs will be necessary to further refine the clinical relevance and validation of these biomarkers.

## 3. Discussion

In this study, an integrative transcriptomic strategy combining mRNA, miRNA, and circRNA data was employed to capture the multilayered regulatory complexity of gastric cancer (GC). The use of eight independent GEO datasets enhanced robustness by reducing dataset-specific variability. This approach aligns with recent multi-omics studies reporting that integrating multiple transcriptome layers improves biomarker reliability and enhances the detection of convergent oncogenic pathways [8,45]. Similarly, comprehensive analyses have shown that multi-omics integration offers superior sensitivity in identifying PI3K/AKT/MAPK alterations compared to single-platform profiling [45]. These findings confirm that multi-layer transcriptomics provides a strong framework for understanding GC biology.

A total of 249 DEGs, 8 DEmiRNAs, and 4 DEcircRNAs were identified, demonstrating a molecular profile characterized by dysregulation of cell cycle progression, extracellular matrix remodeling, and invasion. Upregulated genes such as *COL4A1*, *MMP14*, and *SERPINH1* have been independently associated with GC metastasis and adverse clinical outcomes. MMP14, in particular, is known to drive invasive behavior through matrix degradation [46]. These parallels indicate that the molecular alterations identified here reflect core pathogenic events consistently reported in GC biology. Functional enrichment analyses highlighted key oncogenic pathways, including PI3K–AKT signaling, ECM–receptor interaction, and focal adhesion. These findings correspond with reports showing that GC progression is strongly driven by proliferative and pro-adhesive signaling axes [45,47]. miRNA pathway enrichment further implicated tumor microenvironment remodeling and immune-related regulation, consistent with evidence that miRNAs orchestrate angiogenesis, stromal changes, and immune modulation in GC [48]. Together, these results indicate that GC development is governed by an interplay of coding and non-coding RNA networks converging on major cancer pathways.

The constructed ceRNA network identified regulatory nodes with defined interaction patterns, including IGF2BP3 and *FSCN1*, which participated in specific circRNA–miRNA–mRNA relationships rather than extensive network connectivity. *IGF2BP3* is a well-established m6A reader known to stabilize oncogenic transcripts and promote proliferation in gastric cancer [49,50]. *FSCN1*, a cytoskeletal regulator, has also been shown to enhance motility and metastasis in digestive cancers [51]. Moreover, the circRNAs identified here likely regulate these hubs by acting as miRNA sponges—consistent with reports describing circRNAs as stable ceRNA regulators that modulate miRNA availability [8]. Collectively, these findings suggest that gene-specific and context-dependent regulatory mechanisms, rather than uniform non-linear post-transcriptional networks, may influence gastric cancer progression. Notably, while several hub genes exhibited significant associations with patient survival, *KIF14* did not show a statistically significant prognostic effect, underscoring the heterogeneity of survival patterns among the analyzed genes.

Kaplan–Meier survival analysis revealed 13 hub molecules significantly associated with overall survival. Genes such as *KIF14*, *CENPI*, *PRRX2*, and *TGM2* exhibited strong negative prognostic value, corroborating previous literature. *KIF14* overexpression has been shown to predict advanced TNM stage and reduced survival in GC [52], and its inhibition reduces proliferative and invasive potential in vitro. Similarly, *TGM2* is widely recognized for its role in EMT activation, drug resistance, and metastatic behavior, with elevated expression correlating with aggressive GC phenotypes [53]. These findings collectively support the prognostic validity of the identified hub molecules and highlight their utility as potential biomarkers for patient stratification and risk prediction.

Drug repositioning using the LINCS L1000CDS^2^ framework identified celastrol, pevonedistat, fedratinib, tozasertib, and Withaferin A as promising candidates capable of reversing GC-associated expression profiles. Celastrol has been reported to inhibit NF-κB and PI3K/AKT signaling while suppressing EMT and metastasis in gastrointestinal cancers [54]. Pevonedistat, a NEDD8-activating enzyme inhibitor, induces apoptosis and cell-cycle arrest in solid tumors and has demonstrated antitumor efficacy in preclinical GC models [41]. Fedratinib, although approved for myelofibrosis, targets JAK2/STAT3 pathways known to be aberrant in GC, supporting its potential repurposing [55]. The identification of these compounds suggests that gene-based therapeutic reversal is feasible and highlights drug repurposing as a cost-effective strategy for expanding GC treatment options.

Molecular docking analysis provided structural validation for the repositioned drugs, demonstrating high-affinity interactions with several hub molecules. Celastrol exhibited strong predicted binding to *MMP14*, consistent with experimental studies reporting its ability to suppress matrix metalloproteinases and inhibit metastatic behavior [56,57]. Withaferin A also demonstrated broad binding capacity and is known to induce apoptosis, inhibit cytoskeletal reorganization, and suppress NF-κB activity in cancer cells [58]. The strong concordance between docking predictions and known drug mechanisms highlights the biological plausibility of the repositioned compounds and supports their prioritization for preclinical evaluation in GC. It should be noted that the blind docking strategy employed here does not guarantee ligand binding to experimentally validated active sites. Consequently, the predicted interactions should be interpreted with caution and regarded as candidates for future biochemical and cellular validation rather than definitive proof of target inhibition.

ROC curve analysis demonstrated strong discriminatory performance for several hub molecules, particularly *MMP14* and *TGM2*, whose AUC values approached 1.0. This level of diagnostic accuracy is consistent with previous biomarker studies reporting extracellular matrix–associated genes as sensitive indicators of gastric cancer progression [46,53]. Additionally, *IGF2BP3*, which also demonstrated high AUC values, has been proposed in multiple studies as a diagnostic and prognostic biomarker due to its cancer-specific expression profile [50]. The integration of ROC findings with survival and docking results provides a comprehensive framework for evaluating the dual diagnostic and therapeutic relevance of these hub molecules. Such multi-dimensional validation strengthens their potential utility in precision oncology workflows for GC.

Despite the comprehensive integrative framework employed in this study, several limitations should be acknowledged. First, the analyses are primarily based on publicly available transcriptomic datasets, which, while enabling robust cross-cohort integration and increasing statistical power, may inherently reflect dataset-specific biases related to sample heterogeneity, platform differences, and clinical annotation depth. Although multiple datasets and independent validation strategies were used to mitigate these effects, prospective validation in well-characterized patient cohorts would further strengthen the translational relevance of the findings. Second, the diagnostic performance of hub molecules was assessed using computational metrics such as ROC curve analysis and dimensionality reduction approaches. While these analyses provide strong evidence for discriminatory potential, they do not substitute for functional or clinical validation at the tissue or patient level. Similarly, drug repositioning and molecular docking analyses were conducted to prioritize candidate compounds and explore plausible binding interactions; however, these results should be interpreted as hypothesis-generating rather than definitive proof of therapeutic efficacy. Importantly, the present work was designed as a systems-level discovery study, aiming to identify biologically meaningful and clinically relevant candidates that merit further experimental investigation rather than to deliver immediate clinical interventions.

Future studies should focus on translating the identified hub molecules and candidate drugs into experimental and clinical settings. In particular, validation of key biomarkers such as *MMP14* and *TGM2* using independent patient cohorts, immunohistochemical analyses, or minimally invasive biospecimens would help clarify their diagnostic and prognostic utility. Integration of additional omics layers—such as proteomics, epigenomics, or spatial transcriptomics—may further refine the molecular landscape and capture regulatory mechanisms that are not fully represented at the transcriptional level alone. Moreover, experimental validation of the proposed drug candidates through in vitro functional assays and in vivo disease models will be essential to confirm their mechanistic relevance and therapeutic potential. From a broader perspective, the systems biology framework presented here is readily extensible to other gastrointestinal malignancies or precancerous conditions, supporting its utility as a generalizable platform for biomarker discovery and drug repositioning. Collectively, these future directions underscore the translational promise of the current study while building upon its strengths as a robust, integrative, and hypothesis-driven computational investigation.

## 4. Materials and Methods

### 4.1. Selection of Microarray Datasets

The workflow followed throughout this study is shown in the Graphical Abstract. This study was designed as a fully in silico investigation integrating publicly available transcriptomic, survival, and drug-response datasets. All analyses were performed using multiple independent publicly available datasets rather than a single patient cohort. The transcriptomic datasets for GC and normal gastric tissues were retrieved from the NCBI Gene Expression Omnibus (GEO) database. For mRNA analysis, three expression datasets (GSE152309, GSE65801, and GSE158662) were selected. In addition, three miRNA profiles (GSE30070, GSE28700, and GSE54397) and two circRNA datasets (GSE89143 and GSE184882) were incorporated to enable a comprehensive evaluation of transcriptomic alterations associated with STAD. Detailed information for each dataset is presented in Table 4, including accession IDs, sample sizes, and the technology used.

### 4.2. Identification of DEGs, DEmiRNAs, and DEcircRNAs

All GEO datasets were analyzed using the GEO2R web-based tool [67], which is built upon the limma (Linear Models for Microarray Data) R package. Prior to differential expression analysis, expression values were normalized by quantile normalization to achieve comparable distributions across samples and to minimize technical variability arising from platform- or batch-related effects. The limma workflow includes background correction, log_2_ transformation of expression intensities, and empirical Bayes moderation of variance estimates, an approach that improves statistical reliability.

Differential expression analyses of mRNA, miRNA, and circRNA datasets were analyzed using the GEO2R platform, which integrates R (v4.2.2) and Bioconductor packages including Biobase (v2.58.0), GEOquery (v2.66.0), and limma (v3.54.0) [68]. Benjamini–Hochberg’s correction was employed to control the false discovery rate. For all datasets, DEGs, DEmiRNAs, and DEcircRNAs were identified using a threshold of |log_2_ fold change| > 1 and *p*-value < 0.05. The transcripts with log_2_FC ≤ −1 were categorized as downregulated, while those with log_2_FC ≥ 1 were considered upregulated. The dataset intersection strategy was used to minimize batch-driven artifacts. To determine the overlapping DEGs, DEmiRNAs, and DEcircRNAs across datasets, Venn diagrams were generated separately for each RNA type using the Jvenn interactive tool [69].

### 4.3. Functional Enrichment Assay

Functional enrichment analysis of both the common DEGs and DEmiRNAs was performed using the GeneCodis4 platform [70]. Enrichment was carried out using the Kyoto Encyclopedia of Genes and Genomes (KEGG) [71] and the Gene Ontology (GO) databases [72] to identify significantly overrepresented biological processes, molecular functions, and pathways. The hypergeometric raw *p*-values were corrected with Benjamini/Hochberg FDR, and a significance threshold of *p*-value < 0.05 was applied. In addition to GeneCodis-based annotation, DEmiRNAs were further analyzed using specialized resources to explore their disease relevance and regulatory functions. The Human microRNA Disease Database (HMDD v3) [73] was used to identify known associations between DEmiRNAs and human diseases, while RNADisease [74] provided experimentally supported links to disease phenotypes. Furthermore, TAM 2.0 (Tool for Annotations of Human MicroRNAs) [75] was utilized to classify DEmiRNAs into functional groups, biological pathways, and tissue-specific expression categories. This integrated analytical approach enabled a more comprehensive understanding of the functional roles of both gene- and miRNA-level dysregulation in STAD.

### 4.4. Construction of ceRNA Network

The interactions between the common DEcircRNAs and DEmiRNAs were identified using data retrieved from circAtlas [76], a comprehensive and curated database of human circRNAs. Subsequently, experimentally validated interactions between DEmiRNAs and DEGs were obtained from miRTarBase [77]. These datasets were integrated to construct a competing endogenous RNA (ceRNA) regulatory network by combining DEcircRNA–DEmiRNA and DEmiRNA–DEG interactions. The resulting tripartite ceRNA network (DEGs–DEmiRNAs–DEcircRNAs) was visualized using Cytoscape (v3.9.1) [78].

Topological analysis of the ceRNA network was performed to evaluate its structural and regulatory properties. Network topology was analyzed using the NetworkAnalyzer tool in Cytoscape. Both degree centrality, representing the number of direct interactions associated with each node, and betweenness centrality, reflecting the extent to which a node acts as a bridge connecting different parts of the network, were calculated for all molecular nodes. Based on these metrics, hub molecules were defined as nodes exhibiting high degree and betweenness centrality values, indicating their topologically central positions within the integrated ceRNA regulatory network. This approach was intended to identify molecules with potential regulatory influence based on network structure rather than expression magnitude alone.

In addition, functional enrichment of the target DEGs within the ceRNA network was conducted using Metascape (v3.5.20240901) [79], based on Gene Ontology (GO) terms and KEGG pathway annotations.

### 4.5. Survival Analysis

To identify survival-associated genes, univariate Cox proportional hazards regression analysis was performed. The prognostic significance of each gene was evaluated using Kaplan–Meier (KM) [80] survival curves, comparing overall survival between high-risk and low-risk patient groups based on gene expression levels.

The prognostic strength of each candidate gene was assessed using the log-rank test *p*-value, hazard ratio (HR), and corresponding 95% confidence intervals (CI). Survival analyses focused on the DEGs identified within the ceRNA regulatory network and were conducted using the KM-Plotter online tool. Genes with a log-rank *p*-value < 0.05 were considered statistically significant, indicating potential relevance for patient stratification and clinical prognosis in STAD.

### 4.6. Drug Repositioning

To identify potential therapeutic agents targeting the expression signatures of key DEGs, drug repositioning analysis was performed using the Library of Integrated Network-Based Cellular Signatures (LINCS) L1000CDS^2^ dataset [81]. Candidate small molecules and drugs were prioritized based on their cosine distance scores (1 − cos α), reflecting the degree of inverse correlation between the input gene expression profiles and the molecular signatures induced by the compounds. Higher cosine distance values indicated stronger potential for reversing disease-associated gene expression patterns.

Following transcriptomic prioritization, repositioned compounds were further evaluated according to their U.S. Food and Drug Administration (FDA) approval status, potential clinical limitations, and translational relevance. To support the selection of inhibitory compounds and to assess their biological plausibility, the identified candidates were cross-referenced using multiple publicly available pharmacological databases, including PubChem, the Comparative Toxicogenomics Database (CTD) [82], and DrugBank [83]. In particular, CTD was utilized to examine documented gene–drug interactions and reported inhibitory effects related to cancer-associated pathways. This integrative strategy enabled the systematic prioritization of drug candidates with potential applicability in STAD treatment.

### 4.7. Molecular Docking

Three-dimensional (3D) structural models of the protein products encoded by the target DEGs regulated by DEcircRNAs were retrieved from the Protein Data Bank (PDB) [84] and UniProt databases [85]. To identify candidate small-molecule inhibitors for each prognostic biomarker, data were extracted from the Comparative Toxicogenomics Database (CTD) [82]. The corresponding 3D structures of these compounds were subsequently obtained from the PubChem database [86]. The detailed information for target DEGs, drug molecules, accession IDs, and related references was presented in Table 2 and Table 3. Molecular docking simulations were conducted using the Cavity-detection guided Blind Docking (CB-Dock) platform [87], an automated docking tool that identifies optimal binding cavities and predicts ligand-protein interactions without prior specification of active sites. This docking analysis was conducted as a supportive, hypothesis-generating approach to explore the potential structural compatibility between the identified small molecules and the corresponding protein products, rather than to validate gene expression changes observed in transcriptomic analyses. The docking results were therefore used to provide preliminary insights into possible ligand–protein interaction patterns and binding orientations at the structural level.

### 4.8. ROC Curve Analysis

Receiver operating characteristic (ROC) curve analysis was conducted using the ROC plotter platform [88] to evaluate the diagnostic performance of hub molecules in GC. The area under the curve (AUC) was used as a quantitative measure of classifier accuracy, where 0.5 indicates no discriminatory power, and 1.0 denotes a strong biomarker. In addition to *p*-values, optimal cut-off points were determined to distinguish between responder and non-responder groups. These groups were defined according to drug-response annotations provided by the ROC Plotter platform and its underlying pharmacogenomic datasets. These classifications were derived from the original source studies integrated into ROC Plotter rather than assigned by the authors. For each gene–drug pair, ROC Plotter applied internally optimized expression cut-offs to maximize discrimination between the two response groups, and AUC values were calculated accordingly.

Hub molecules achieving AUC values above 0.70 were considered to display significant discriminatory capacity and were prioritized as potential biomarkers, particularly in relation to repositioned drug candidates identified in this study. Furthermore, ROC analyses were performed separately for the five repositioned drugs and 13 hub molecules assessed in this study.

### 4.9. Validation of Transcriptomic and Proteomic Expression Levels of Candidate Hub Molecules In Silico

Gene expression levels of the prioritized hub molecules *MMP14* and *TGM2* in normal gastric tissues and gastric cancer samples were evaluated using the TNMplot online platform [89] (https://tnmplot.com/analysis/, access date: 27 September 2025). TNMplot integrates uniformly processed transcriptomic data derived from Gene Expression Omnibus (GEO), The Cancer Genome Atlas (TCGA), and Genotype-Tissue Expression (GTEx) databases, enabling direct and statistically robust comparisons between normal and tumor tissues. Differential expression between groups was assessed using the statistical framework implemented by the platform, and corresponding *p*-values were reported as provided by TNMplot.

Protein expression patterns of *MMP14* and *TGM2* were examined using immunohistochemical (IHC) data retrieved from the Human Protein Atlas (HPA) database [90] (https://www.proteinatlas.org/, access date: 28 September 2025). Representative IHC images from gastric tumor tissues and normal glandular tissues were evaluated qualitatively based on staining intensity (weak, moderate, strong) and the proportion of positively stained cells, as annotated in the HPA repository. HPA IHC data are based on independent tumor and normal tissue samples and are not derived from matched patient pairs. All protein expression data were derived from antibody-based profiling and were used to support transcriptomic findings at the protein level. In HPA immunohistochemistry data, protein expression levels are evaluated based on staining intensity and the quantity of positive cells, where “Quantity” indicates the proportion of immunoreactive tumor cells.

## 5. Conclusions

This study presents a comprehensive integrative multi-omics framework to delineate circRNA-mediated ceRNA regulatory networks in gastric cancer and to prioritize clinically relevant biomarkers and therapeutic candidates. By jointly analyzing mRNA, miRNA, and circRNA expression profiles across multiple independent cohorts, we minimized platform-specific bias and increased biological robustness, enabling the identification of convergent regulatory programs underlying gastric tumorigenesis. Through network-based systems biology approaches combined with survival modeling, we uncovered 13 prognostically significant hub molecules, including *MMP14* and *TGM2*, whose dysregulation was consistently supported at both transcriptomic and proteomic levels. The concordance between gene-level alterations, patient outcomes, and protein expression patterns underscores the translational relevance of these molecules as potential biomarkers for risk stratification and disease monitoring in gastric cancer.

Transcriptomics-guided drug repositioning coupled with structure-based molecular docking further highlighted five candidate compounds (celastrol, fedratinib, pevonedistat, tozasertib, and withaferin A) as promising modulators of the identified regulatory hubs. The integration of survival analyses, docking simulations, and drug-response–oriented ROC profiling provides a multilayered validation strategy that elevates confidence in both the mechanistic plausibility and clinical applicability of these candidates. Importantly, the predictive ROC analyses performed in drug-treated gastric cancer models move beyond conventional diagnostic assessments by linking hub gene expression to therapeutic responsiveness, thereby positioning *MMP14* and *TGM2* as potential transcriptomic indicators of treatment sensitivity. This aspect adds a potential translational dimension to the study and supports the use of ceRNA-informed biomarkers in guiding precision oncology strategies. Collectively, this work demonstrates how integrative RNA-level network modeling, when coupled with pharmacogenomic interrogation and in silico structural validation, can bridge molecular discovery and therapeutic hypothesis generation in gastric cancer. The methodological pipeline described here is readily extendable to other gastrointestinal malignancies and precancerous conditions, offering a generalizable platform for biomarker discovery and rational drug repositioning. While experimental and prospective clinical validation will be required to translate these findings into practice, the present study establishes a solid foundation for future mechanistic investigations and targeted therapeutic development in gastric cancer.

## Figures and Tables

**Table 1:** Significant hub molecules identified from the ceRNA core network.

Gene Name	Description	Function	Uniprot ID	Inhibitor’s Name	PubChem ID	Reference
***IGF2BP3***	Insulin-like growth factor 2 mRNA-binding protein 3	RNA binding protein, post-translational regulator, and mRNA stabilizer, m6A reader	O00425	Acetaminophen	1983	[14]
***CDK1***	Cyclin-dependent kinase 1	Cell cycle control; modulates the centrosome of mitotic division and promotes G2-M in the interphase stage	P06493	Belinostat	6918638	NA
***ASCL2***	Achaete-scute homolog 2	Transcription factor	Q99929	Bezafibrate	39042	NA
***CTHRC1***	Collagen triple helix repeat-containing protein 1	Negative regulator of collagen matrix deposition	Q96CG8	Calcitriol	5280453	[15]
***COL4A1***	Collagen alpha-1(IV) chain	Inhibits angiogenesis, tumor formation, cell proliferation, migration, and tube formation anti-angiogenic activity	P02462	Acetaminophen	1983	[16,17]
***KIF14***	Kinesin-like protein KIF14	Takes part in cell division, cytokinesis, cell proliferation, and apoptosis.	Q15058	Acrylamide	6579	[18]
***MCUB***	Calcium uniporter regulatory subunit MCUb, mitochondrial	An inhibitor of the MCU channel that controls calcium entry into mitochondria.	Q9NWR8	Chlordecone	299	[19]
***PRXX2***	Paired mesoderm homeobox protein 2	Effects on the healing of cutaneous wounds	Q99811	Chlordecone	299	[20]
***SERPINB5***	**Serpin B5**	Tumor suppressor, blocks invasion and metastatic tumors	P36952	Genistein	5280961	[21]
***CLDN1***	Claudin-1	Key components of tight junctions that control epithelial barrier permeability.	O95832	Amitrole	1639	[22]
***COL12A1***	Collagen alpha-1(XII) chain	Interacts with other collagen types; associated with surface fibrils.	Q99715	Alitretinoin	449171	[23]
***MMP14***	Matrix metalloproteinase-14	An endopeptidase that breaks down multiple elements of the extracellular matrix	P50281	Acetamide	178	[24]
***CKAP2***	Cytoskeleton-associated protein 2	Stabilizing microtubules and regulating aneuploidy, cell cycle, and cell death	Q8WWK9	Azathioprine	2265	[25]
***FSCN1***	**Fascin**	Actin-binding protein and organization of filaments and cytoskeleton units	Q16658	Acrylamide	6579	[26]
***SERPINH1***	Serpin H1	Binds collagen and may act as a chaperone during its biosynthesis.	P50454	Acetaminophen	1983	NA
***EXO1***	**Exonuclease 1**	DNA exonucleases and the repair of DNA mismatch	Q9UQ84	Afuresertib	46843057	[27]
***CENPF***	Centromere protein F	Takes part in the kinetochore function and chromosome segregation	P49454	Abrine	160511	[28]
***SGO2***	**Shugoshin 2**	Works with PPP2CA to maintain centromeric cohesin in meiosis I	Q562F6	Abrine	160511	[29]
***TGM2***	Protein-glutamine gamma-glutamyltransferase 2	Catalyzes the formation of covalent bonds between various amines and crosslinking proteins	P21980	Atrazine	2256	[30]
***PBXIP1***	Pre-B-cell leukemia transcription factor-interacting protein 1	Regulates PBX transcription factors by blocking PBX1-HOX DNA binding and E2A-PBX1 activity. Links ESR1 to microtubules, affecting its signaling	Q96AQ6	Abrine	160511	[31]
***CENPI***	Centromere protein I	Plays a central role in chromosome segregation and mitotic progression	Q92674	Acetaminophen	1983	[32]

**Table 2:** Repositioned drug candidates and their utilization, mode of action, and FDA approval statuses.

Perturbation	PubChem ID	1—Cosα	Indication	MOA	Approval Status	Reference
**Celastrol**	122724	16.702	Used to treat inflammation and autoimmune diseases, including rheumatoid arthritis, systemic lupus erythematosus, nephritis, and asthma. It is an antioxidant and anti-inflammatory drug.	- An EC 5.99.1.3 [DNA topoisomerase (ATP-hydrolysing)] inhibitor - An Hsp90 inhibitor	Investigational	[33]
**At-7519**	11338033	16.727	Used for selectively inhibiting CDKs, leading to cell cycle arrest, programmed cell death (apoptosis), and suppression of tumor cell growth.	- Inhibitor of specific Cyclin Dependent Kinases (CDKs) - Antineoplastic agent	Investigational	[34]
**Buparlisib**	16654980	17.478	Used to treat melanoma, metastases, lung cancer, solid tumors, and breast cancer.	- Selective inhibitor of PI3K - Antineoplastic agent - Penetrate the blood–brain barrier	Investigational	[35]
**Withaferin-A**	265237	17.327	Antitumor and anti-inflammatory effect, Used in pancreatic cancer and breast cancer cells.	- Inhibits nuclear factor-κb (NF-κb) - Activation of ıκb kinase via a thiol alkylation-sensitive redox mechanism, - Apoptosis inducer - Targets heat shock protein 90	Investigational, formerly FDA-approved	[36]
**Elesclomol**	300471	17.859	Used to treat melanoma	- Pro-apoptotic activity - Induce ROS activity - Antitumor activity	Investigational	[37]
**Fedratinib**	16722836	17.473	Used to treat adults with intermediate-2 or high-risk primary or secondary myelofibrosis, including cases following polycythemia vera or essential thrombocythemia.	- Inhibitor of JAK2 kinease and FLT3	Approved	[38]
**Nocodazole**	4122	16.745	binds to β-tubulin, inhibiting microtubule polymerization and causing G2/M cell cycle arrest, making it useful for mitosis studies and cell synchronization.	- Antineoplastic agent - Tubulin modulator - Antimitotic and a microtubule-destabilizing agent	Investigational	[39]
**Parthenolide**	7251185	17.518	Potential in treating leukemia and inhibiting NF-κB signaling.	- Anticancer chemotherapeutic, anti-metastatic, anti-angiogenic, anti-inflammatory, and antinociceptive activities	Approved	[40]
**Pevonedistat**	16720766	16.781	Used to treat lymphoma, solid tumors, multiple myeloma, Hodgkin lymphoma, and metastatic melanoma	- Antineoplastic activity - Inhibitor of Nedd8 activity enzyme (NAE)	Investigational	[41]
**Rigosertib**	6918736	16.671	Used to treat myelodysplastic syndromes (MDS), refractory anemia with excess blasts (RAEB), cancer, hepatoma, and other neoplasms	- Bind to Ras-binding domain (RBD) and induce cell cycle arrest, apoptosis, and proliferation of tumors.	Investigational	[42]
**Sertraline**	68617	17.117	Used to treat depression, anxiety disorders, panic disorder, obsessive–compulsive disorder (OCD), and post-traumatic stress disorder	- Antidepressant of the serotonin reuptake inhibitor - Cytochrome P450 2D6 Inhibitor	Approved	[43]
**Tozasertib**	5494449	17.830	Used to treat acute myeloid leukemia (AML), chronic myeloid leukemia (CML), and other hematologic malignancies	- Inhibitor of Aurora Kinase - Induce Apoptosis - Inhibits FLT3 kineases	Investigational	[44]

**Table 3:** ROC curve analysis results in AUC values and associations between hub molecules and repositioned drug candidates.

Hub Molecules	Tozasertib	Pevonedistat	Fedratinib
	AUC Value	AUC Value	AUC Value
***IGF2BP3***	0.625	0.667	0.5
***COL4A1***	0.625	0.762 *	0.5
***KIF14***	0.625	0.81 *	0.55
***MCUB***	0.75 *	0.619	0.65
***CENPI***	0.625	0.571	0.65
***PRRX2***	0.625	0.714 *	0.55
***CLDN1***	0.625	0.738	0.75 *
***MMP14***	0.625	1 *	0.75 *
***CKAP2***	0.625	0.714 *	0.6
***FSCN1***	0.625	0.524	0.75 *
***SERPINH1***	0.625	0.619	0.7 *
***SGO2***	0.5	0.667	0.55
***TGM2***	1 *	0.714 *	0.8 *

* AUC > 0.70 values are highlighted with an asterisk (*) as indicative of significant classification performance.

**Table 4:** Selection of datasets specific to GC transcriptomic profiling.

Detected RNA Types	GEO Series ID	Platform	Tissue	Samples	Study Design	References
**Messenger RNA**	GSE152309	Illumina NextSeq 500	GC	15	5 N/5 GC	[59]
	GSE65801	Agilent SurePrint G3 Human Microarray	GC	64	32 N/32 GC	[60]
	GSE158662	Agilent Human lncRNA + mRNA Array	GC	6	3 N/3 GC	[61]
**MicroRNA**	GSE30070	Agilent Human miRNA Microarray	GC	132	34 N/98 GC	[62]
	GSE28700	Agilent Human miRNA Microarray	GC	44	22 N/22 GC	[63]
	GSE54397	Agilent Human miRNA Microarray	GC	32	8 N/8 GC	[64]
**Circular RNA**	GSE89143	Agilent Arraystar Human CircRNA Microarray	GC	6	3 N/3 GC	[65]
	GSE184882	Agilent Arraystar Human CircRNA Microarray	GC	8	4 N/4 GC	[66]

## Data Availability

All datasets used in this study are publicly available at the Gene Expression Omnibus (GEO) Database. https://www.ncbi.nlm.nih.gov/geo/browse/?view=series, access date 1 August 2025.

## Abbreviations

The following abbreviations are used in this manuscript:

GC	Gastric Cancer
STAD	Stomach Adenocarcinoma
circRNA	Circular RNA
mRNA	Messenger RNA
miRNA	MicroRNA
ceRNA	Competing Endogenous RNA
WHO	World Health Organization
ncRNAs	Non-Coding RNAs
GO	Gene Ontology
KM	Kaplan–Meier
GEO	Gene Expression Omnibus
TCGA	The Cancer Genome Atlas
EGA	European Genome-Phenome Archive
LINCS	Library of Integrated Network-Based Cellular Signatures
FDA	U.S. Food and Drug Administration
CTD	Comparative Toxicogenomics Database
PDB	Protein Data Bank
CB-Dock	Cavity-detection guided Blind Docking
AUC	The area under the curve
ROC	Receiver operating characteristic
